# Supplementary material for: Robust Automated Mouse Micro-CT Segmentation Using Swin UNEt TRansformers
Source: Bioengineering (Basel). 2024 Dec 11;11(12):1255. doi: 10.3390/bioengineering11121255 (PMC11673508; doi:10.3390/bioengineering11121255)
Supplement: Supplementary file 1 [file bioengineering-11-01255-s001.zip › bioengineering-3357461-supplementary.pdf]

Robust Automated Mouse Micro-CT Segmentation Using Swin UNEt TRansformers

Supplementary Material

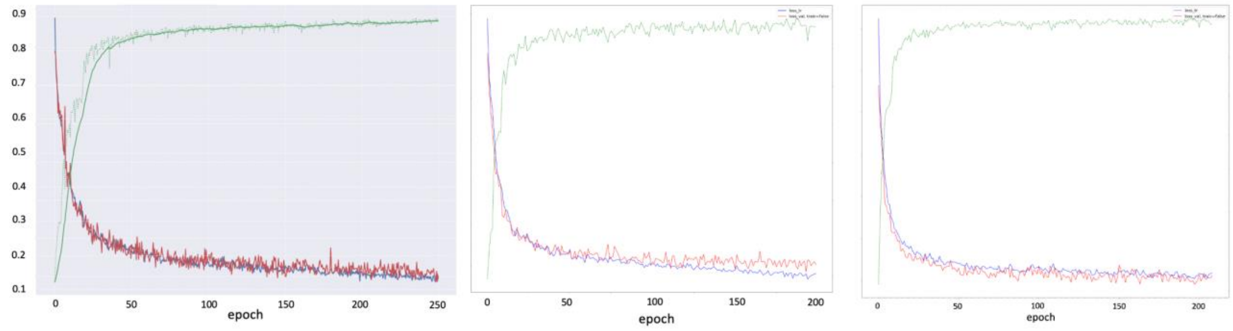

Figure S1: This figure shows the training loss (blue), validation loss (red), and pseudo-Dice score (green) at different epochs. The results for Swin-UNETR, 3D nnU-Net, and 2D AIMOS are presented from left to right.

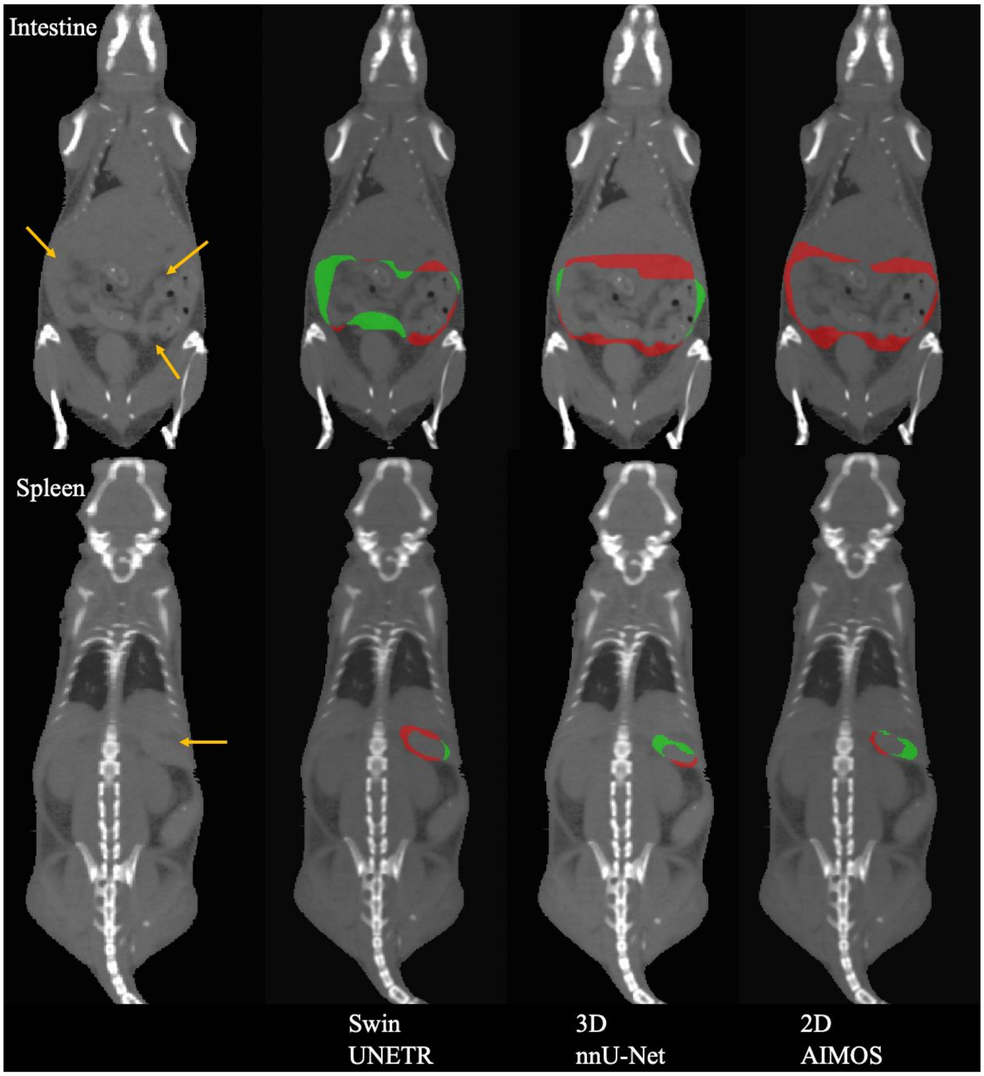

Figure S2: Intestine (top) and spleen (bottom) segmentation analysis with under-segmentation highlighted in green and over-segmentation highlighted in red. The figure shows a representative median-scored case from the IHCT test set in a coronal view. The columns represent segmentation results from Swin-UNETR, 3D nnU-Net, and 2D AIMOS, respectively. This result shows that native micro-CT images exhibit poor abdominal contrast, leading to both UNet models over-segmenting the intestine. Swin UNETR shows reduced over-segmentation but presents more false negative volumes. Yellow arrows highlight ambiguous regions contributing to segmentation challenges.

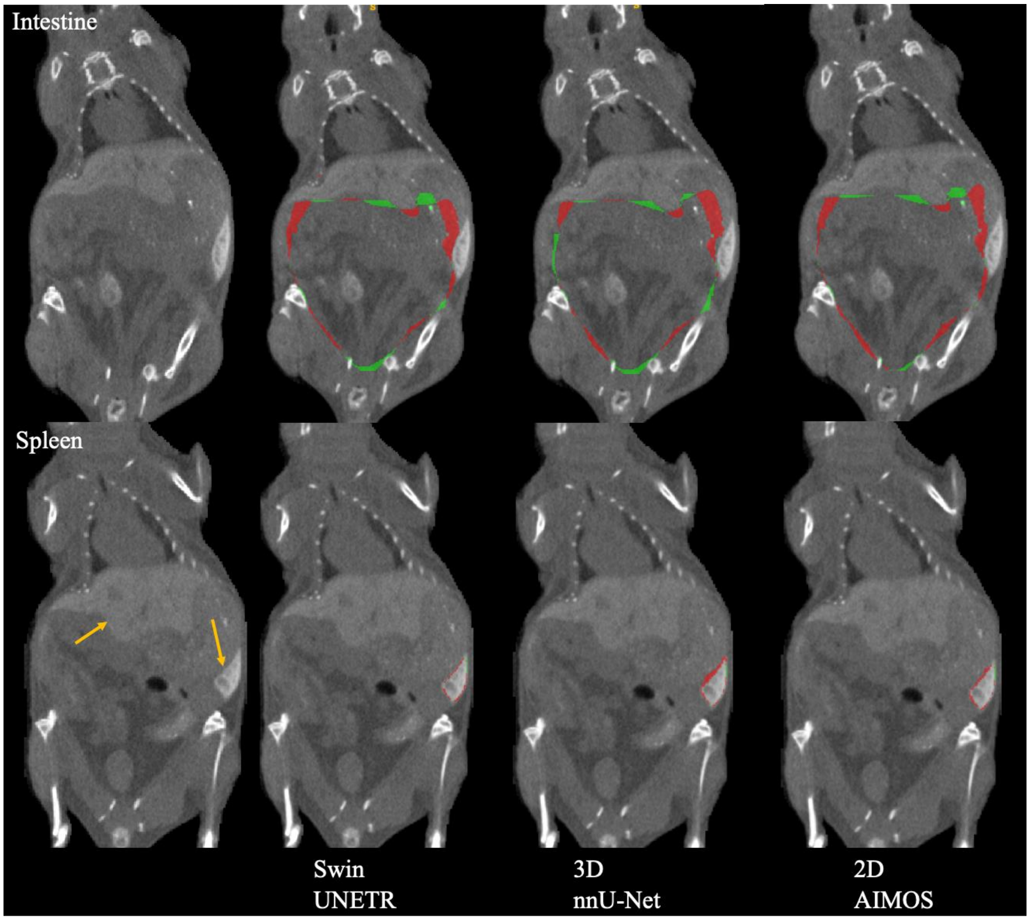

Figure S3: Intestine (top) and spleen (bottom) segmentation analysis with under-segmentation highlighted in green and over-segmentation highlighted in red. The figure shows a representative median-scored case from the CECT test set in a coronal view. Compared to Supplementary Material Figure S2, the use of a contrast agent significantly improves the boundary quality of the liver and spleen (highlighted by yellow arrows), reducing ambiguity in model interpretation. While all three models show over-segmentation on the spleen towards the intestine side, Swin-UNETR mitigates this issue with fewer false positive voxels.
